# Association between Iron Intake and Progression of Knee Osteoarthritis

**DOI:** 10.3390/nu14081674

**Published:** 2022-04-18

**Authors:** Limin Wu, Haibo Si, Yi Zeng, Yuangang Wu, Mingyang Li, Yuan Liu, Bin Shen

**Affiliations:** Department of Orthopaedics, Orthopedic Research Institute, West China Hospital, Sichuan University, Chengdu 610041, China; wulimin@stu.scu.edu.cn (L.W.); sihaibo@schscu.cn (H.S.); zengyigd@126.com (Y.Z.); wuyuangang23@scu.edu.cn (Y.W.); limingyang199606@stu.scu.edu.cn (M.L.); yihaoliuyuan@163.com (Y.L.)

**Keywords:** osteoarthritis, iron, intake, iron overload, progression, cohort

## Abstract

Background: Iron overload is drawing attention in the development of knee osteoarthritis (OA). To identify the modifiable risk factors for iron-related pathological conditions, we examined the association between iron intake and the risk of knee OA progression. Methods: A total of 1912 participants in the Osteoarthritis Initiative (OAI), aged 45–79 years and with at least one knee radiographic OA at baseline, were identified and were followed up to 6 years. The iron and other nutrient intake was measured by the validated Block Brief 2000 Food Frequency Questionnaire. The outcome measures were by radiographic progression on the basis of the Kellgren–Lawrence (KL) grade and the joint-space-narrowing (JSN) score. The association between the iron intake and the knee OA progression was examined by Cox proportional hazards models and restricted cubic spline (RCS) regression. Results: Among the study participants, 409 participants experienced KL-grade progression, and 684 participants experienced JSN-score progression within 6 years. Overall, the association between iron intake and the risk of KL-grade progression followed a U shape (*p* for nonlinearity < 0.001). The risk of KL-grade progression was significantly lower in participants with iron intakes of <16.5 mg/day (per mg/day: adjusted hazard ratio (HR), 0.75; 95% CI (confidence interval), 0.64–0.89), and it was higher in those with iron intakes ≥16.5 mg/day (per mg/day: HR, 1.20; 95% CI, 1.04–1.38). Consistently, when the iron intake was assessed as deciles, compared to those in Deciles 3–5 (10.9–23.3 mg/day), the risk of KL-grade progression was higher for Deciles 1–2 (≤10.9 mg/day: HR, 1.57; 95% CI, 1.17–2.10) and for Deciles 6–10 (>23.3 mg/day: adjusted HR, 1.60; 95% CI, 1.19–2.16). Similar U-shaped relations were found for iron intake with the risk of JSN-score progression (*p* for nonlinearity = 0.035). Conclusions: There was a U-shaped association between the iron intake and the progression of knee OA, with an inflection point at about 16.5 mg/day, and minimal risk from 10.9 to 23.3 mg/day of iron intake. An appropriate iron intake was advisable for knee OA, whereas excessive or deficient iron intake increased the risk of knee OA progression.

## 1. Introduction

Osteoarthritis (OA) is a chronic disabling disease that is characterized by progressive articular cartilage damage coupled with deterioration in subchondral bone and osteophyte formation [1]. The pain, stiffness, deformity, and dysfunction of the joints that are caused by knee OA are the most frequent causes of disability and the impairment of quality of life [2]. Since the controversy around the effects of structure-modifying treatment, and the limited efficacy of surgical intervention in early- or middle-stage knee OA, comprehensive disease management, such as behavioral interventions, education, and exercise, is considered to be the first-line treatment by the guidelines [3]. In the last decade, behavioral interventions, with diet modifications, have been suggested to be effective at reducing OA incidence or progression. Different dietary patterns and macronutrient compositions, such as Western dietary patterns [4], saturated fat [5], and dietary fiber [6], are considered to be associated with the incidence of knee OA.

In contrast to these macronutrient compositions, the risks of micronutrients are not well understood, and especially minerals that may affect knee OA through metabolic processes and immune functions [7]. Of these, iron is an essential trace element for many biological metabolic reactions in chondrocytes, such as redox reaction, DNA synthesis, and cellular respiration in chondrocytes [8,9]. However, the window of beneficial iron concentrations is narrow. The association between iron overload and OA pathogenesis is drawing attention, as excess free iron catalyzes reactive-oxygen-species (ROS) production and leads to oxidant-mediated cellular damage [10]. Clinical and experimental evidence suggests a considerable association between OA pathogenesis and iron overload. Increased levels of iron crystal deposition in synovial-lining cells and iron concentrations in synovial fluid have been observed in knee OA patients [11,12], and higher levels of serum ferritin were linked to the radiographic severity of the knee OA in an American cohort [13]. Moreover, a preclinical experiment has demonstrated that systemic iron overload, which was induced by the weekly intraperitoneal injection of iron dextran to guinea pigs for 4 weeks, resulted in cellular iron accumulation in the knee joint, and led to the loss of the chondrocyte and cartilage proteoglycan contents [14]. Excess iron levels within OA joint tissues have been established as a risk factor for the progression of OA [15]. However, the levels of these iron biomarkers are not only dependent on the iron status, but are also influenced by inflammation, liver diseases, and other metabolic conditions [16]. The question of whether iron dietary intake is linked to knee OA progression in humans still requires further clarification.

For the prevention of iron-related pathological conditions, it is, therefore, important to identify the modifiable risk factors for iron deficiency or overload, especially since, unlike other minerals, the iron levels in the body are controlled only by absorption [17]. The mechanism of iron excretion is an unregulated process that is characterized by the rapid turnover and excretion of enterocytes [17]. Comprehensive analyses of the relationship between iron intake and OA progression remain to be reported. To fill these gaps in the knowledge, we conducted a longitudinal cohort study to investigate the association between iron intake and the risk of knee OA progression.

## 2. Method

### 2.1. Study Design and Population

We performed a cohort study by using prospectively collected data from the Osteoarthritis Initiative (OAI) database. The OAI is a multicenter longitudinal study of 4796 men (41.5%) and women aged 45–79 years, with or at risk of knee OA, who were recruited from 2004 to 2006. The participants were followed annually by centrally trained rheumatologists or radiologists at each clinical center, about 97% of them had ≥1 follow-up visit, and the visit adherence was not influenced by the baseline characteristics. The study was fully compliant with the NIH guidelines, and all the participants provided informed consent. The detailed OAI protocol is described elsewhere [18].

To assess the knee OA progression, we included participants with at least one knee with radiographic OA at baseline, which is defined by a Kellgren–Lawrence (KL) grade ≥ 2, on the basis of the central reading of a standardized fixed-flexion radiographic. The index knee from each participant was used, and if the participant had two eligible knees, the one with a KL grade = 3 was selected as the index knee; if both knees had the same KL grade, the knee with the higher Western Ontario and McMaster Universities Osteoarthritis Index (WOMAC) knee-pain score was selected as the index knee; if both knees had the same WOMAC pain score, we randomly selected one as the index knee by using a random number generator. Participants without radiographic OA (KL grade = 0 or 1 at baseline) were excluded, as were those with severe radiographic OA (KL grade = 4), as they were less likely to experience further radiographic progression. We also excluded participants with missing or implausible total daily calorie intakes (<800 or >4200 kcal for men; <500 or >3500 kcal for women) [19]. Finally, 1912 participants were included and were followed up at 12, 24, 36, 48, and 72 months.

### 2.2. Assessment of Dietary Nutrient Intake

The dietary intake of minerals and other nutrients was estimated at baseline by using the validated dietary assessment tool: the Block Brief 2000 Food Frequency Questionnaire (FFQ), of seventy items [20]. For each item, the participants’ typical food and average consumption over the past year were assessed according to the predetermined categories, which ranged from “never” to “every day”. Food-consumption data were converted to minerals, and the other nutrient intakes were calculated by using the nutrient-composition values developed in the Second National Health and Nutrition Examination Survey [21]. In addition, in order to assess the mineral intake from nutritional supplements, specific sections of the FFQ were used to record the average daily amount of 30 days of a vitamin/supplement-combination intake. The reported information, in conjunction with the food composition, was used to calculate the participants’ daily minerals (iron, sodium, potassium, calcium, zinc, and magnesium) and the intake of other main nutrients (calories, fat, carbohydrate, and protein).

### 2.3. Measurement of Nondietary Covariates for Knee OA

On the basis of the previous evidence of the potential determinants of the structural OA progression, the following baseline characteristics were extracted: age; sex; race; education; family income; the Physical Activity Scale for the Elderly (PASE) score (2–445); the body mass index (BMI); the nonsteroidal anti-inflammatory drug (NSAID) use; the baseline KL grade; and the baseline joint-space-narrowing (JSN) score.

### 2.4. Outcome Identification

The knee OA progression was assessed by using two endpoints: (1) The KL-grade progression; and (2) The JSN-score progression. Knee radiographs were taken at baseline and follow-up visits by using similar acquisition and reading protocols. The KL grade and JSN scores were assessed by the same readers. The KL-grade progression was defined as an increase in the KL grade ≥ 1 at a follow-up visit, compared with that at baseline. The JSN scores were evaluated separately in the medial and lateral tibiofemoral compartments, and the JSN-score progression was defined as an increase in the JSN score ≥ 1 in either the medial or lateral tibiofemoral as the JSN progression. If a knee underwent a knee replacement, as in other studies, we assigned it a KL grade of 4 and a JSN score of 3 at the first visit after the replacement because the radiographs that showed the knee replacement did not allow for an assessment of the KL grade or JSN score [22,23]. The date of the follow-up replacement was defined as the nearest OAI visit to the follow-up after the knee replacement (radiographs and/or medical records).

### 2.5. Statistical Analysis

The participants were classified into ten groups, according to the deciles of the iron intake. Comparisons between categorical variables by iron groups were evaluated by using χ^2^ statistics, and ANOVA tests were used to evaluate the continuous variables. The incidence rates of the OA progression, expressed as per 1000 person-years, were calculated as the number of OA-progression cases divided by the person-years of follow-up. The relation of the iron intake to the OA progression was estimated by using Cox proportional hazards models (hazard ratio (HR) and a 95% CI), with and without adjustments for the age, sex, BMI, PASE score, NSAID use, baseline KL grade, baseline JSN score, mineral intake (iron, sodium, potassium, calcium, zinc, and magnesium), as well as the intake of other main nutrients (calories, fat, carbohydrate, and protein). A threshold analysis of the relationship between iron intake and OA progression was performed by using a 2-piecewise Cox regression model with a smoothing function. The inflection point was determined by the R package “segmented”, using a likelihood-ratio test and the bootstrap resampling method. Restricted cubic spline (RCS) Cox regression was conducted, with 4 knots (5th, 35th, 65th, and 95th percentiles as the knots, and the inflection point was set as the reference), to test for linearity and to explore the shape of the dose–response relation of the iron intake and the OA progression. Additionally, to test the robustness of the primary findings, we conducted sensitivity analyses for each outcome by the baseline knee KL grade and the baseline score. R software, version 4.1.2 (http://www.R-project.org, accessedon 13 March 2022), was used for all data analyses.

## 3. Result

**Figure 1:**
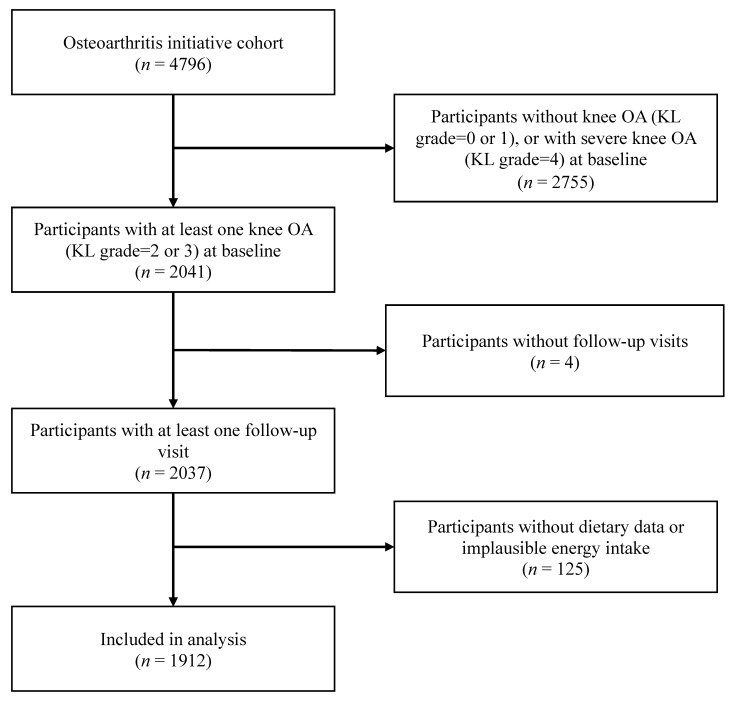
Flowchart of screening-eligible subjects. OA = osteoarthritis; KL grade = Kellgren–Lawrence grade.

A total of 1912 participants with 1912 knees from the OAI were included in the present study. Figure 1 describes the eligible participants that were included in the final analyses. The average age of the study population was 62.1 ± 9.0 years, and 59% were female. The mean iron intake was 24.1 ± 16.8 mg/day. More than 89.5% of the participants underwent all follow-up visits, and no significant difference in the rate of loss to follow-up was observed among the decile groups of each iron intake pattern. The baseline characteristics across deciles of the total iron intake are summarized in Table 1. Participants with higher iron intakes were more likely to have lower BMIs, higher education, higher PASE scores, and higher amounts of calorie intake.

**Figure 2:**
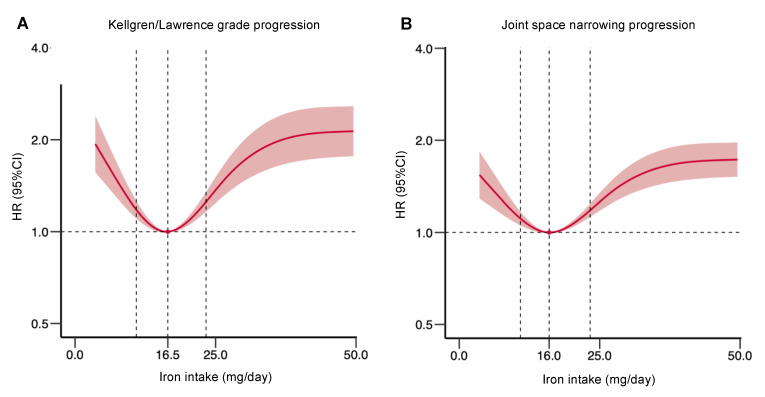
Relation of iron intake to risk of (**A**) KL-grade and (**B**) JSN-score progression. Dashed vertical lines represent inflection points and relative minimal risk thresholds of 10.9 mg/day and 23.3 mg/day (Deciles 3–5). Estimates adjusted for age, sex, BMI, PASE score, NSAID use, baseline KL grade, baseline JSN score, mineral intake (iron, sodium, potassium, calcium, zinc, and magnesium), as well as intake of other main nutrients (calories, fat, carbohydrate, and protein). The *p* values for overall association and the *p* values for nonlinearity were less than 0.05 for all outcomes. HR = hazard ratio.

A total of 409 participants experienced KL-grade progression during the 6-year follow-up. Overall, the association between the iron intake and the risk of KL-grade progression followed a U shape (Figure 2A; *p* for nonlinearity < 0.001). In the threshold effect analysis, the risk of KL-grade progression was significantly lower with the increments of iron intake (per mg/day: adjusted HR, 0.75; 95% CI, 0.64–0.89) in the participants with iron intakes < 16.5 mg/day, and was higher with the increments of iron intake (per mg/day: adjusted HR, 1.20; 95% CI, 1.04–1.39) in the participants with iron intakes ≥ 16.5 mg/day (Table 2). When the iron intake was assessed as deciles, compared with Deciles 1–2, the participants in Deciles 3–5 have a lower risk of KL-grade progression (Table 3), whereas the participants in Deciles 6–10 have a relatively higher risk of KL-grade progression. Therefore, we combined the iron intake into three categories, compared with those in Deciles 3–5 (10.9–23.3 mg/day). The risk of KL-grade progression was higher for Deciles 1–2 (<10.9 mg/day: adjusted HR, 1.57; 95% CI, 1.17–2.10) and for Deciles 6–10 (≥23.3 mg/day: adjusted HR, 1.60; 95% CI 1.19–2.16).

Moreover, 684 participants experienced JSN-score progression during the 6-year follow-up. Overall, the association between the iron intake and the risk of JSN-score progression also followed a U shape (Figure 2B; *p* for nonlinearity = 0.035). In the threshold effect analysis, the risk of JSN-score progression was significantly lower with the increments of iron intake (per mg/day: adjusted HR, 0.86; 95% CI, 0.75–0.97) in the participants with iron intakes < 16.0 mg/day, and it was higher with the increments of iron intake (per mg/day: adjusted HR, 1.10; 95% CI, 1.03–1.16) in the participants with iron intakes ≥ 16.0 mg/day (Table 2). When the iron intake was assessed as deciles, compared with Deciles 1–2, the participants in Deciles 3–5 have a lower risk of JSN-score progression, whereas the participants in 6–10 have a relatively higher risk of JSN-score progression (Table 4). Therefore, we combined the iron intake into three categories, and, compared with those in Deciles 3–5 (10.9–23.3 mg/day), the risk of JSN-score progression was higher for Deciles 1–2 (<10.9 mg/day: adjusted HR, 1.40; 95% CI, 1.12–1.76) and for Deciles 6–10 (≥23.3 mg/day: adjusted HR, 1.37; 95% CI 1.08–1.73).

We additionally investigated sensitivity analyses for each outcome by the KL grade and JSN score at baseline. The results were not significant (*p* values ranged from 0.23 to 0.95).

## 4. Discussion

In this large prospective longitudinal study with a 6-year follow-up period, we found that there was a U-shaped association between the total iron intake and the knee OA progression, with an inflection point at about 16.5 mg/day, and minimal risk from 10.9 to 23.3 mg/day of total iron intake. These findings were independent of the effect of the major confounders and they remained stable in the sensitivity analyses.

Our findings are in line with other studies that have found associations between dietary patterns, and that have provided some new insights into this field. First, among the participants with iron intakes < 16.5 mg/day, one plausible mechanism for the risk of the OA progression being significantly lower with the increments of iron intake may be that iron is an essential element of various metabolic processes in chondrocytes, including DNA synthesis, the secretion of the extracellular matrix, and oxygen transport [17]. The lack of normal iron levels in chondrocytes could cause mitochondrial damage or dysfunction that results in a large accumulation of ROS and nitric oxide (NO), which, in turn, induces significant metabolic disturbances, an enhanced inflammatory response, cell death, and other typical features of OA progression. Second, among the participants with iron intakes ≥ 16.5 mg/day, the underlying mechanism for the risk of OA progression being significantly higher with the increments of iron intake may include the fact that excessive intake leads to elevated iron levels in chondrocytes, which compromises cellular homeostasis, triggers oxidative stress and mitochondrial dysfunction, as well as ferroptosis, apoptosis, or autophagy, and ultimately results in synovial inflammation and articular cartilage degeneration [10]. Therefore, the maintenance of an appropriate iron intake is essential to the maintenance of cartilage homeostasis and to the inhibition of the progression of OA.

The potential adverse health effects of a high-iron status have been reported in recent decades. In a 10-year longitudinal study, a high-iron status was associated with an increased risk of death [24], and, in some national studies, serum ferritin was positively associated with the risk of diabetes [25,26]. Our study adds knee OA progression to the list of potential high-iron-related health problems. In the National Health and Nutrition Examination Survey (NHANES), the iron intake declined by ~6.6 and ~9.5% for male and female adults, respectively, from 1999 to 2018, with 18.4% of adult females, and 4.6% of adult males, not meeting the daily iron intake standards that are recommended by the U.S. College of Medicine, Food and Nutrition Board, and the trend was still worsening, which may be associated with an increase in iron deficiency anemia and the associated mortality in the U.S. population [27]. Moreover, in the China Health and Nutrition Survey (CHNS), an average iron intake that ranged from 22.1 to 26.1 mg/day was reported between 1991 and 2004 (CHNS) [28]. There has been a substantial improvement in the iron status of the Chinese population, as CHNS data show a 53% decrease in the prevalence of anemia, from 20.8% in 2002, to 9.7% in 2012 [29]. Therefore, for those with or at risk of knee OA, timely policy adjustments may be needed in different countries, and the beneficial window of iron intake (10.9 to 23.3 mg/day) should be considered. Otherwise, the burden of knee OA may increase further because the bioavailability of iron will further decrease/increase with the ongoing decrease in the iron concentration reduction in the majority (62.1%) of American food products, and with the increased intake of animal foods in the Chinese diet.

Although an investigation or detailed discussion of the specific foods that are associated with iron intake is beyond the scope of this research, it is noteworthy that iron-rich foods include red meat, whole-grain cereals, and dark-green vegetables. In addition, recent experimental results suggest that the antioxidants, and, in particular, vitamins C and E, may counteract some of the deleterious effects of dietary iron on diabetes and breast cancer by preventing lipid peroxidation [30,31,32]. However, the results of this longitudinal study do not imply that the population with knee OA will benefit from dietary advice, although our study shows that adjustments for the other major nutrients did not materially alter the findings, and recent studies demonstrate that iron supplementation and/or suggestions of dietary changes resulted in a decrease in the prevalence of iron deficiency among Swiss blood donors [33]. The role of suggestions for dietary change should be further investigated first.

This study has several limitations that should be addressed. First, because of the multidimensionality of diets, other nutrients that coexist in foods that are rich in iron are examined. To reduce this possibility, we controlled for several dietary and nondietary covariates, and particularly for the BMI and other mineral intake, in order to reduce the confounding effects. Secondly, the iron intake was assessed by self-reported dietary intakes, which may be slightly less reliable than more objective methods, although a validated FFQ was used. However, this should lead to decreased statistical power, which makes these findings all the more notable. Finally, the dietary information was only obtained at baseline, and the change in the participants’ dietary habits during the follow-up years could not be updated. However, the FFQ measures long-term diet, which is less likely to change significantly within 6 years, on the basis of previous studies [4,6], and such measurement error would bias the results towards the null [34].

In summary, there was a U-shaped association between iron intake and the progression of knee OA, with an inflection point at about 16.5 mg/day, and a minimal risk at 10.9 to 23.3 mg/day of iron intake. An appropriate iron intake was advisable for knee OA, whereas excessive or deficient iron intake increased the risk of knee OA progression.

## Figures and Tables

**Table 1:** Population characteristics by categories of dietary iron intake.

Characteristics	Total	Iron Intake (mg/day)										*p* Value
		Decile 1	Decile 2	Decile 3	Decile 4	Decile 5	Decile 6	Decile 7	Decile 8	Decile 9	Decile 10	
		(≤7.7)	(7.7–10.9)	(10.9–14.0)	(14.0–18.2)	(18.2–23.3)	(23.3–25.7)	(25.7–27.8)	(27.8–30.7)	(30.7–35.6)	(>35.6)	
*N*		195	189	190	195	192	198	181	190	191	191	
Age (year)	62.1 ± 9.0	61.9 ± 9.4	61.6 ± 9.0	61.0 ± 8.9	59.3 ± 8.8	61.2 ± 8.4	63.3 ± 8.9	64.3 ± 8.6	64.1 ± 9.0	62.1 ± 9.0	62.0 ± 8.9	<0.001
Sex (female, %)	59	62.1	57.1	56.3	45.6	63.0	72.2	65.7	60.0	53.9	54.5	<0.001
Race (%)												<0.001
White	77.2	66.7	74.6	70.5	74.9	72.9	81.8	86.2	83.7	83.2	78.5	
African American	20.4	29.7	22.8	26.8	23.6	24.5	17.2	10.5	12.6	15.2	20.4	
Other	2.4	3.6	2.6	2.7	1.5	2.6	1.0	3.3	3.7	1.5	1.0	
Education (%)												0.017
≤High School	17.7	24.6	22.8	20.5	21.0	16.1	13.1	8.8	14.7	14.1	20.4	
College	45.5	44.1	46.0	45.8	44.1	44.3	47.0	49.2	47.4	45.0	42.4	
>College	36.8	31.3	31.2	33.2	34.9	39.6	39.9	42.0	37.9	40.8	37.2	
Missing	0.1	0	0	0.5	0	0	0	0	0	0	0	
Family income (%)												0.235
<25 k	14.2	14.4	16.9	17.4	13.8	12.0	11.6	9.4	12.1	17.3	17.3	
25–50 k	25.4	25.6	25.9	27.9	23.1	25.5	28.8	23.2	27.4	19.4	26.7	
50–100 k	34.3	34.9	33.3	34.2	37.9	38.5	28.3	40.3	31.6	35.1	28.8	
≥100 k	19.5	16.4	16.4	15.8	22.1	17.2	21.2	19.9	20.5	23.0	22.0	
Missing	6.7	8.7	7.4	4.7	3.1	6.8	10.1	7.2	8.4	5.2	5.2	
PASE score	157.9 ± 8.1	149.2 ± 80.8	159.2 ± 78.2	159.9 ± 80.0	162.0 ± 83.2	166.3 ± 78.5	146.1 ± 78.9	155.7 ± 73.5	146.8 ± 78.2	163.2 ± 83.3	170.1 ± 94.5	0.038
BMI (kg/m^2^)	29.6 ± 4.8	30.2 ± 4.8	30.3 ± 5.0	30.2 ± 5.3	30.6 ± 5.0	29.5 ± 4.7	29.1 ± 4.8	28.6 ± 4.4	28.5 ± 4.6	29.4 ± 4.4	29.3 ± 5.0	<0.001
KL grade (%)												0.890
2	62.0	60.5	57.1	64.2	63.1	65.1	63.6	63.0	62.1	62.3	59.2	
3	38.0	39.5	42.9	35.8	36.9	34.9	36.4	37.0	37.9	37.7	40.8	
JSN score (%)												0.820
0	23.5	19.0	21.7	21.6	24.1	30.2	25.3	25.4	24.2	21.5	22.5	
1	38.6	41.5	36.0	43.2	39.0	34.9	38.4	37.6	37.9	40.8	36.6	
2	37.9	39.5	42.3	35.3	36.9	34.9	36.4	37.0	37.9	37.7	40.8	
NSAID use (%)	27.0	29.2	28.6	23.7	30.9	26.6	21.7	24.9	27.9	26.8	29.8	0.595
Dietary intake												
Calories (1000 kcal/day)	1.5 ± 0.7	1.0 ± 0.3	1.3 ± 0.3	1.5 ± 0.5	1.8 ± 0.6	1.3 ± 0.7	1.2 ± 0.3	1.3 ± 0.3	1.6 ± 0.4	1.8 ± 0.5	1.8 ± 0.7	<0.001
Fat (g/day)	56.3 ± 26.5	40.0 ± 15.6	51.3 ± 18.8	60.7 ± 24.4	70.0 ± 28.8	50.8 ± 32.6	44.9 ± 17.9	49.4 ± 19.2	60.5 ± 23.9	70.0 ± 24.9	66.3 ± 32.8	<0.001
Carbohydrate (g/day)	170.6 ± 70.2	112.3 ± 41.0	147.6 ± 43.0	182.8 ± 62.7	203.5 ± 72.6	152.8 ± 84.7	135.0 ± 43.0	153.0 ± 45.9	187.3 ± 57.2	218.0 ± 57.4	214.7 ± 86.1	<0.001
Protein (g/day)	62.0 ± 25.6	38.0 ± 11.9	53.1 ± 14.9	62.8 ± 18.4	73.7 ± 24.0	55.4 ± 30.0	49.8 ± 16.0	59.34 ± 18.2	68.9 ± 19.7	78.5 ± 20.4	81.4 ± 35.8	<0.001
Sodium (g/day)	1.9 ± 0.8	1.2 ± 0.4	1.6 ± 0.5	2.0 ± 0.6	2.3 ± 0.7	1.7 ± 0.9	1.5 ± 0.5	1.7 ± 0.5	2.1 ± 0.6	2.4 ± 0.7	2.4 ± 1.0	<0.001
Potassium (g/day)	2.5 ± 1.0	1.5 ± 0.5	2.1 ± 0.6	2.6 ± 0.8	2.9 ± 0.9	2.3 ± 1.1	2.1 ± 0.6	2.5 ± 0.7	2.9 ± 0.8	3.3 ± 0.8	3.3 ± 1.4	<0.001
Calcium (g/day)	1.2 ± 0.6	0.7 ± 0.5	0.8 ± 0.4	1.0 ± 0.6	1.0 ± 0.4	1.1 ± 0.5	1.3 ± 0.5	1.3 ± 0.6	1.4 ± 0.5	1.5 ± 0.6	1.5 ± 0.6	<0.001
Zinc (mg/day)	20.0 ± 14.1	6.7 ± 8.4	9.2 ± 8.2	12.8 ± 11.4	14.2 ± 7.7	20.4 ± 9.8	24.1 ± 11.4	25.0 ± 10.9	27.4 ± 12.3	27.4 ± 10.7	33.0 ± 19.2	<0.001
Magnesium (mg/day)	303.4 ± 116.8	146.7 ± 44.5	207.8 ± 55.8	264.6 ± 72.0	298.6 ± 82.8	286.8 ± 80.5	293.7 ± 52.2	335.5 ± 68.0	372.4 ± 81.1	420.3 ± 89.9	412.4 ± 155.2	<0.001

*N* = number of participants; BMI = body mass index; PASE = Physical Activity Scale for the Elderly; JSN = joint space narrowing; KL = Kellgren–Lawrence; NSAIDs = nonsteroidal anti-inflammatory drugs.

**Table 2:** Threshold effect analyses of iron intake on the risk of knee OA progression using 2-piecewise regression models.

Iron Intake (mg/day)	Unadjusted Model		Adjusted Model †	*p* Value
	HR (95%CI)	*p* Value	HR (95%CI)	
KL grade				
<16.5	0.76 (0.64–0.89)	0.002	0.75 (0.64–0.89)	0.001
≥16.5	1.14 (1.02–1.29)	0.025	1.20 (1.04–1.38)	0.010
JSN score				
<16.0	0.85 (0.75–0.97)	0.014	0.86 (0.75–0.97)	0.021
≥16.0	1.09 (1.04, 1.15)	<0.001	1.10 (1.03–1.16)	0.002

† Adjusted for age, sex, BMI, PASE score, NSAID use, baseline KL grade, baseline JSN score, mineral intake (iron, sodium, potassium, calcium, zinc, and magnesium), as well as intake of other main nutrients (calories, fat, carbohydrate, and protein). JSN = joint space narrowing; KL = Kellgren–Lawrence; HR = hazard ratio; CI = confidence interval.

**Table 3:** The association between iron intake and the risk of KL-grade progression.

Iron Intake (mg/day)	*N*	Cases (Incidence Rate) §	Unadjusted Models	*p* Value	Adjusted Models †	*p* Value
			HR (95% CI)		HR (95% CI)	
Deciles						
≤7.7	195	52 (4.7)	Ref		Ref	
7.7–10.9	189	42 (3.8)	0.82 (0.55–1.23)	0.345	0.81 (0.54–1.21)	0.304
10.9–14.0	190	32 (2.7)	0.60 (0.39–0.94)	0.025	0.61 (0.39–0.95)	0.027
14.0–18.2	195	24 (2.0)	0.44 (0.27–0.72)	0.001	0.45 (0.28–0.73)	0.001
18.2–23.3	192	33 (2.8)	0.62 (0.40–0.96)	0.033	0.71 (0.45–1.12)	0.141
23.3–25.7	198	41 (3.4)	0.75 (0.50–1.13)	0.164	0.77 (0.49–1.22)	0.262
25.7–27.8	181	44 (4.1)	0.89 (0.60–1.34)	0.591	1.01 (0.64–1.59)	0.955
27.8–30.7	190	45 (4.0)	0.88 (0.59–1.30)	0.522	1.02 (0.65–1.60)	0.940
30.7–35.6	191	43 (3.8)	0.83 (0.56–1.25)	0.375	0.93 (0.58–1.50)	0.780
>35.6	191	53 (4.8)	1.04 (0.71–1.53)	0.826	1.21 (0.77–1.90)	0.403
Categories						
Deciles 1–2 (≤10.9)	384	94 (4.2)	1.64 (1.23–2.20)	0.001	1.57 (1.17–2.10)	0.003
Deciles 3–5 (10.9–23.3)	577	89 (2.5)	Ref		Ref	
Deciles 6–10 (>23.3)	951	226 (4.0)	1.58 (1.24–2.02)	<0.001	1.60 (1.19–2.16)	0.002

† Adjusted for age, sex, BMI, PASE score, NSAID use, baseline KL grade, baseline JSN score, mineral intake (iron, sodium, potassium, calcium, zinc, and magnesium), as well as intake of other main nutrients (calories, fat, carbohydrate, and protein). The § incident rate is presented as per 1000 person-years of follow-up. KL = Kellgren–Lawrence; HR = hazard ratio; CI = confidence interval.

**Table 4:** The association between dietary iron intake and the risk of JSN-score progression.

Iron Intake (mg/day)	*N*	Cases (Incidence Rate) §	Unadjusted Models	*p* Value	Adjusted Models †	*p* Value
			HR (95% CI)		HR (95% CI)	
Deciles						
≤7.7	195	81 (8.6)	Ref		Ref	
7.7–10.9	189	69 (7.3)	0.88 (0.64–1.21)	0.431	0.90 (0.65–1.24)	0.514
10.9–14.0	190	58 (5.6)	0.70 (0.50–0.98)	0.040	0.72 (0.51–1.00)	0.053
14.0–18.2	195	51 (4.8)	0.60 (0.42–0.85)	0.004	0.61 (0.43–0.87)	0.005
18.2–23.3	192	56 (5.3)	0.66 (0.47–0.93)	0.017	0.74 (0.53–1.04)	0.089
23.3–25.7	198	69 (6.5)	0.80 (0.58–1.10)	0.169	0.88 (0.64–1.21)	0.432
25.7–27.8	181	71 (7.5)	0.92 (0.67–1.27)	0.611	1.03 (0.75–1.43)	0.837
27.8–30.7	190	74 (7.8)	0.93 (0.68–1.28)	0.671	1.04 (0.76–1.43)	0.802
30.7–35.6	191	73 (7.7)	0.93 (0.68–1.27)	0.638	1.00 (0.73–1.38)	0.988
>35.6	191	82 (8.9)	1.05 (0.77–1.43)	0.744	1.10 (0.81–1.49)	0.546
Categories						
Deciles 1–2 (≤10.9)	384	150 (7.9)	1.44 (1.15–1.79)	0.001	1.40 (1.12–1.76)	0.004
Deciles 3–5 (10.9–23.3)	577	165 (5.2)	Ref		Ref	
Deciles 6–10 (>23.3)	951	369 (7.6)	1.41 (1.18–1.70)	<0.001	1.37 (1.08–1.73)	0.009

† Adjusted for age, sex, BMI, PASE score, NSAID use, baseline KL grade, baseline JSN score, mineral intake (iron, sodium, potassium, calcium, zinc, and magnesium), as well as intake of other main nutrients (calories, fat, carbohydrate, and protein). The § Incident rate is presented as per 1000 person-years of follow-up. KL = Kellgren–Lawrence; HR = hazard ratio; CI = confidence interval.

## Data Availability

All data are from the OAI public database: https://nda.nih.gov/oai (accessed on 13 March 2022). **Consent for Publication:** Not applicable. (All data are from the OAI public database: https://nda.nih.gov/oai) (accessed on 13 March 2022).

## Abbreviations

KL grade	Kellgren–Lawrence grade
JSN score	Joint-space-narrowing score
OA	Osteoarthritis
BMI	Body mass index
PASE	Physical Activity Scale for the Elderly
NSAIDs	Nonsteroidal anti-inflammatory drugs
FFQ	Block Brief 2000 Food Frequency Questionnaire
